# Analysis of the effect of HDAC inhibitors on the formation of the HIV reservoir

**DOI:** 10.1128/mbio.01632-24

**Published:** 2024-08-13

**Authors:** Lijun Ling, Manse Kim, Andrew Soper, Martina Kovarova, Rae Ann Spagnuolo, Nurjahan Begum, Jennifer Kirchherr, Nancie Archin, Diana Battaglia, Dave Cleveland, Angela Wahl, David M. Margolis, Edward P. Browne, J. Victor Garcia

**Affiliations:** 1 International Center for the Advancement of Translational Science, University of North Carolina at Chapel Hill, Chapel Hill, North Carolina, USA; 2 Division of Infectious Diseases, Department of Medicine, University of North Carolina at Chapel Hill, Chapel Hill, North Carolina, USA; 3 Center for AIDS Research, University of North Carolina at Chapel Hill, Chapel Hill, North Carolina, USA; 4 Department of Microbiology, University of Alabama at Birmingham, Birmingham, Alabama, USA; 5 UNC HIV Cure Center, University of North Carolina at Chapel Hill, Chapel Hill, North Carolina, USA; 6 Center for AIDS Research, University of Alabama at Birmingham, Birmingham, Alabama, USA; 7 Department of Microbiology and Immunology, School of Medicine, University of North Carolina at Chapel Hill, Chapel Hill, North Carolina, USA; University of Pittsburgh School of Medicine, Pittsburgh, Pennsylvania, USA

**Keywords:** histone deacetylase inhibitors, human immunodeficiency virus, reservoir, latency

## Abstract

The HIV reservoir is more dynamic than previously thought with around 70% of the latent reservoir originating from viruses circulating within 1 year of the initiation of antiretroviral therapy (ART). In an *ex vivo* model system of HIV latency, it was reported that early exposure to class I histone deacetylase (HDAC) inhibitors might prevent these more recently infected cells from entering a state of stable viral latency. This finding raises the possibility that co-administration of HDAC inhibitors at the time of ART initiation may prevent the establishment of much of the HIV reservoir. Here, we tested the effects of the HDAC inhibitors suberoylanilide hydroxamic acid (SAHA) and panobinostat co-administered at the time of ART initiation on the formation of the viral reservoir in HIV-infected humanized mice. As previously shown, SAHA and panobinostat were well tolerated in humanized mice. Unexpectedly, co-administration of SAHA resulted in an increase in the frequency of CD4^+^ cells carrying HIV DNA but did not alter the frequency of cell-associated HIV RNA in HIV-infected, ART-treated humanized mice. Co-administration of panobinostat did not alter levels of cell-associated HIV DNA or RNA. Our *in vivo* findings indicate that co-administration of HDAC inhibitors initiated at the same time of ART treatment does not prevent recently infected cells from entering latency.

**IMPORTANCE:**

Current antiretroviral therapy (ART) does not eradicate cells harboring replication-competent HIV reservoir. Withdrawal of ART inevitably results in a rapid viremia rebound. The HIV reservoir is more dynamic than previously thought. Early exposure to class I histone deacetylase (HDAC) inhibitors inhibit these more recently infected cells from entering a state of stable viral latency in an *ex vivo* model of latency, raising the possibility that co-administration of HDAC inhibitors at the time of ART initiation may reduce much of the HIV reservoir. Here, we tested the effects of the HDAC inhibitors suberoylanilide hydroxamic acid or panobinostat during ART initiation on the formation of the viral reservoir in HIV-infected humanized mice. Our *in vivo* study indicates that in contrast to *in vitro* observations, the co-administration of HDAC inhibitors at the same time of ART initiation does not prevent recently infected cells from entering latency.

## INTRODUCTION

The main barrier to an HIV cure is the persistence of the latent viral reservoir during suppressive antiretroviral therapy (ART); treatment withdrawal inevitably results in rapid viral rebound (1, 2). In clinical trials, latency-reversing agents (LRAs) have shown promise in inducing HIV expression (3–6), but thus far, combinations of LRAs and immunotherapies have failed to significantly reduce the latent reservoir (7–13).

Studies show that persistent, latent HIV or SIV infection can be established even if ART is initiated within hours of infection (14–16). Therefore, trying to prevent the establishment of the latent reservoir after the initial infection was thought to be impractical. However, recent studies indicate that the latent reservoir that is present during viremia is more dynamic than previously thought (17–19), as approximately 70% of the latent viral reservoir is genetically linked to viruses circulating within 1 year of ART initiation in people living with HIV who have maintained years of virologic suppression (17). This discovery suggests that alterations in CD4^+^ T cell biology that occur with ART initiation may also allow proviruses to enter into and remain in a prolonged state of latency, leading to the hypothesis that interventions might be developed to disrupt the stabilization of proviruses in latency and reduce the size of the latent reservoir.

Acetylation and deacetylation of histone proteins are correlated with transcriptional activation and repression, respectively. The maintenance of HIV latency is driven, at least in part, by histone deacetylases (HDACs), a family of chromatin-associated enzymes that facilitate the deacetylation of histone proteins and thus restrict the access of transcription factors to viral DNA (20). HDAC inhibitors have been shown to induce HIV expression in clinical trials (3, 5, 6, 21). Among HDAC inhibitors, suberoylanilide hydroxamic acid (SAHA) and panobinostat are the HDAC inhibitors that have been investigated most extensively as HIV latency reversal agents (3, 4, 21). A recent study indicates that HDACs play a critical role in initiating latency, and in an *ex vivo* model of HIV latency, HDAC inhibitors could prevent recently infected cells from entering latency (22). Here, we evaluated the effect of SAHA and panobinostat on the levels of HIV RNA and DNA in blood and tissues when treatment was started at the same time of ART initiation in HIV-infected humanized mice. The results from our *in vivo* analysis show that administration of SAHA results in an increase in the levels of cell-associated HIV DNA. Interestingly, similar treatment with panobinostat does not result in an increase in the levels of cell-associated HIV DNA.

## RESULTS

### Sustained *in vitro* and *in vivo* release of SAHA from an *in situ* forming implant

In contrast to humans, SAHA has a relatively short half-life of just ~0.75 hours in mice (23). Therefore, in order to provide a sustained level of drug throughout the course of the experiment, a long-acting (LA) delivery system was created by formulating SAHA as a biodegradable *in situ* forming implant (ISFI) essentially as previously described for other drugs (24–28). *In vitro* and *in vivo* experiments were then conducted to evaluate the release properties of the resulting SAHA-ISFI formulation.

**Fig 1:**
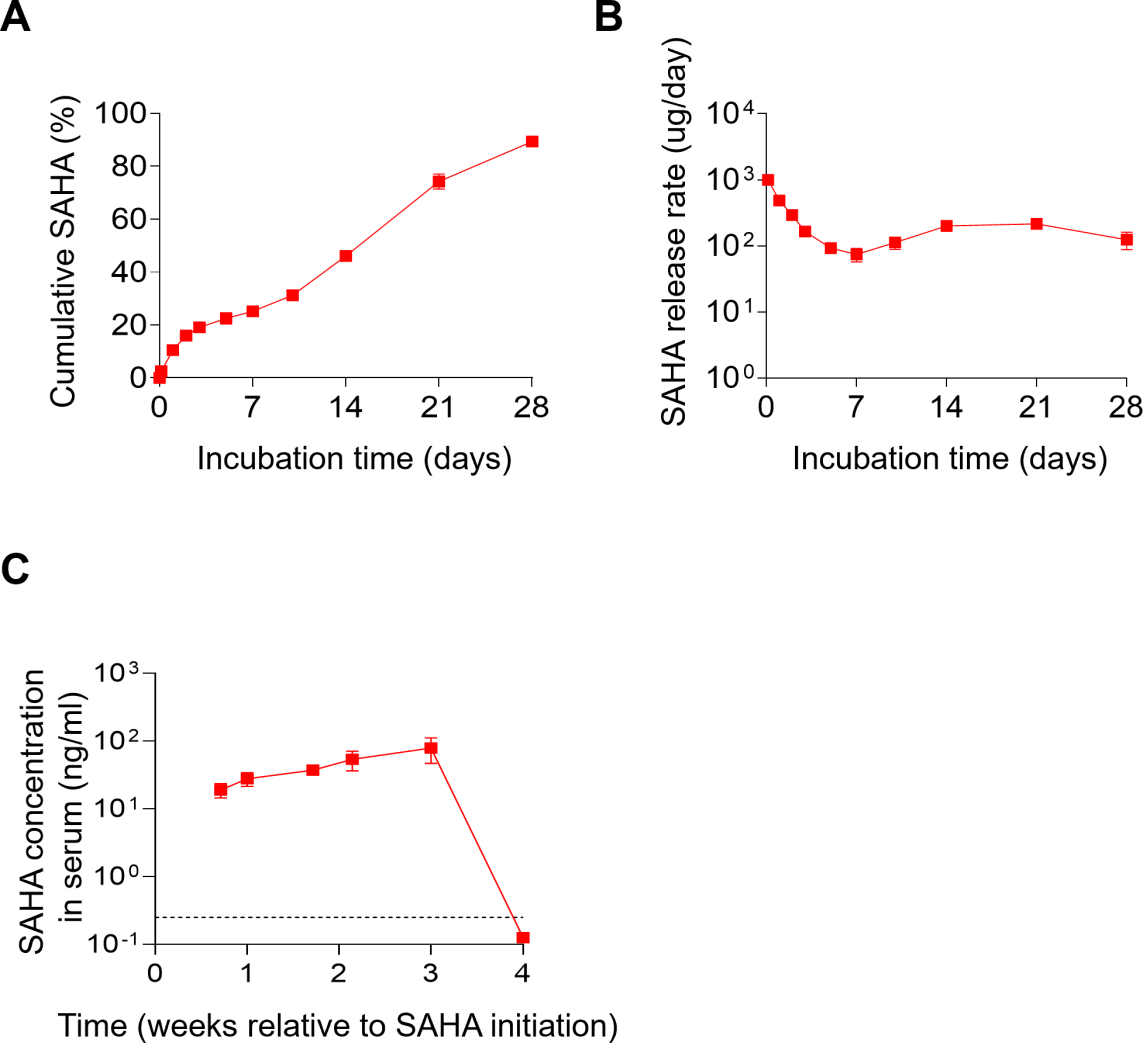
*In vitro-* and *in vivo*-sustained release of SAHA is achieved using an ISFI. (**A**) *In vitro* daily cumulative SAHA release from the SAHA-ISFI formulation (30 µL) (*n* = 3). (**B**) *In vitro* daily SAHA release from the SAHA-ISFI formulation in A (*n* = 3). (**C**) *In vivo* serum concentration of SAHA in BALB/c mice (*n* = 4) after a single subcutaneous injection (50 µL) of the SAHA-ISFI. Data are expressed as mean ± SEM.

The *in vitro* release profile of the formulation was evaluated using 30 µL of SAHA-ISFI in 10 mL of phosphate-buffered saline (PBS). This implant was used to evaluate cumulative drug release over time and the release rate of SAHA from the ISFI. An initial burst of 10.5% (day 1) was observed (Fig. 1A). After the first day, SAHA was released in a linear fashion, with a cumulative total of 89.3% of SAHA released from the long-acting formulation by day 28 (Fig. 1A). On average, the release rate was 159.7 µg/day from day 2 to day 28 (Fig. 1B).

To analyze the *in vivo* pharmacokinetics, a single dose (50 µL) SAHA-ISFI was administered subcutaneously into BALB/c mice. Since no significant changes were observed in mouse body weight at any timepoint after the administration of SAHA-ISFI relative to the baseline (day 0) (Table S1), the serum drug levels were not normalized for body weight. Serum samples were collected and analyzed longitudinally following SAHA-ISFI administration. As shown in Fig. 1C, there was a sustained release of SAHA (34.5 ± 5.5 ng/mL; mean ± SEM) from the ISFI into serum for 3 weeks. No SAHA was detectable in serum, and no implants were found in week 4 in all SAHA-ISFI-treated mice, indicating that SAHA was completely released from the ISFI at this timepoint.

### SAHA-ISFI treatment administered starting at the time of ART initiation increases the levels of cell-associated HIV DNA but does not alter the levels of cell-associated HIV RNA in peripheral blood cells

**Fig 2:**
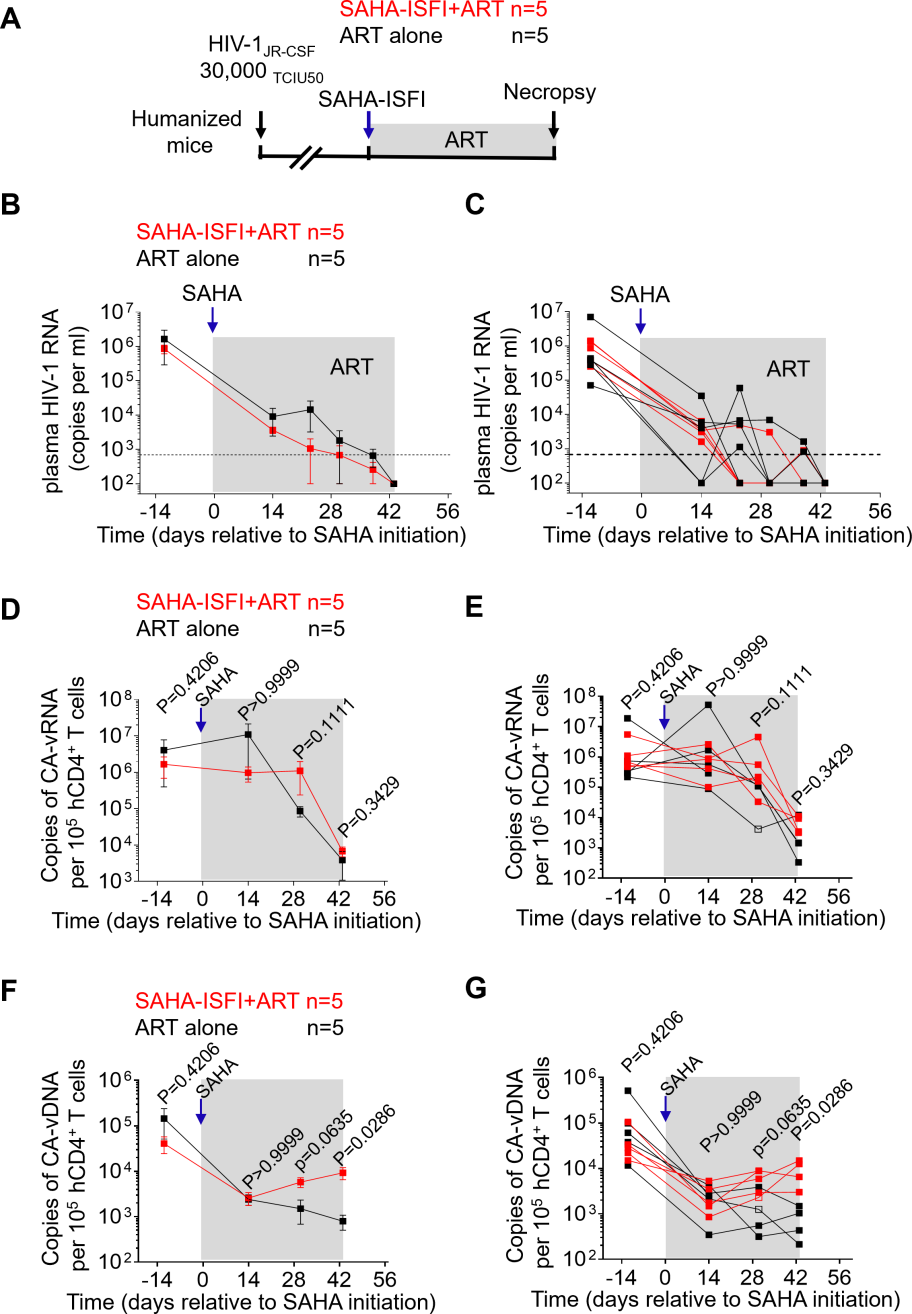
SAHA-ISFI treatment at the same time of ART initiation increases the levels of cell-associated HIV DNA but does not alter the levels of cell-associated HIV RNA in the peripheral blood cells of HIV-infected ART-treated humanized mice. (**A**) Experimental design. Humanized mice were exposed to HIV on day −61 and then were treated with either SAHA-ISFI plus ART or ART alone on day 0. ART was administered via FTC (1,500 mg/kg), TDF (1,560 mg/kg), and RAL (600 mg/kg). Longitudinal assessment of plasma viral loads as groups (**B**) and for individual animals (**C**) over the course of the experiment. The plasma viral load limit of detection (693 copies/mL) is indicated by a dashed line. Longitudinal detection of cell-associated (CA) viral RNA as groups (**D**) and for individual animals (**E**) in HIV-infected mice after treatment with SAHA-ISFI plus ART (red, *n* = 5) or ART alone (black, *n* = 5). Longitudinal detection of CA viral DNA as groups (**F**) and for individual animals (**G**) in HIV-infected mice after treatment with SAHA-ISFI plus ART (red, *n* = 5) or ART alone (black, *n* = 5). One animal from the SAHA-ISFI plus ART group was found dead on day 40. One animal from the ART alone group became ill and was euthanized on day 24. Data were collected for those two animals from our analysis of peripheral blood until the last timepoint before death. Blue arrows indicate the time of the SAHA-ISFI administration. Shaded gray areas depict ART treatment. Data are expressed as mean ± SEM. Open symbols in panels E and G indicating the limit of detection. Statistical analyses were performed using unpaired two-sided Mann-Whitney *U*-tests. Statistical significance was considered when *P* < 0.05.

Humanized mice were infected intravenously with HIV-1_JR-CSF_ (30,000 TCIU50) on day −61 (Fig. 2A) and then treated with either SAHA-ISFI plus ART or ART alone on day 0. ART was administered via irradiated chow containing emtricitabine (FTC), tenofovir disoproxil fumarate (TDF), and raltegravir (RAL) as previously described (29–32). As a control, a group of HIV-infected mice were administered ART alone. Plasma viral load was measured longitudinally following HIV infection and during treatment. There were no significant differences in plasma viral loads between the SAHA plus ART group and the ART alone group throughout the course of treatment (Fig. 2B and C).

To determine the effect of SAHA plus ART treatment on the size of the peripheral blood HIV reservoir compared with ART alone, we measured the levels of cell-associated HIV RNA and DNA in peripheral blood cells longitudinally. No significant differences in the levels of cell-associated HIV RNA were observed between mice treated with SAHA plus ART and those treated with ART alone (Fig. 2D and E). Throughout the course of the experiment, there were no significant differences in the levels of cell-associated HIV DNA between mice treated with SAHA plus ART and mice treated with ART alone (Fig. 2F and G). However, at the end of the experiment (day 43), peripheral blood cell-associated HIV DNA levels were significantly higher (*P* = 0.0286) in SAHA plus ART-treated mice (9,243 ± 2,722 copies, mean ± SEM) compared with the mice treated with ART alone (791 ± 289 copies; mean ± SEM) (Fig. 2F and G). The HIV RNA/DNA ratio was 6.7 times lower in peripheral blood cells from animals that received SAHA when compared with animals that only received ART. These results demonstrate that SAHA treatment during the time of ART initiation results in an increase in the levels of cell-associated HIV DNA but does not alter the levels of cell-associated HIV RNA in the peripheral blood of HIV-infected, ART-treated humanized mice.

### SAHA treatment administered starting at the time of ART initiation increases the levels of cell-associated HIV DNA but does not alter systemic levels of cell-associated HIV RNA

**Fig 3:**
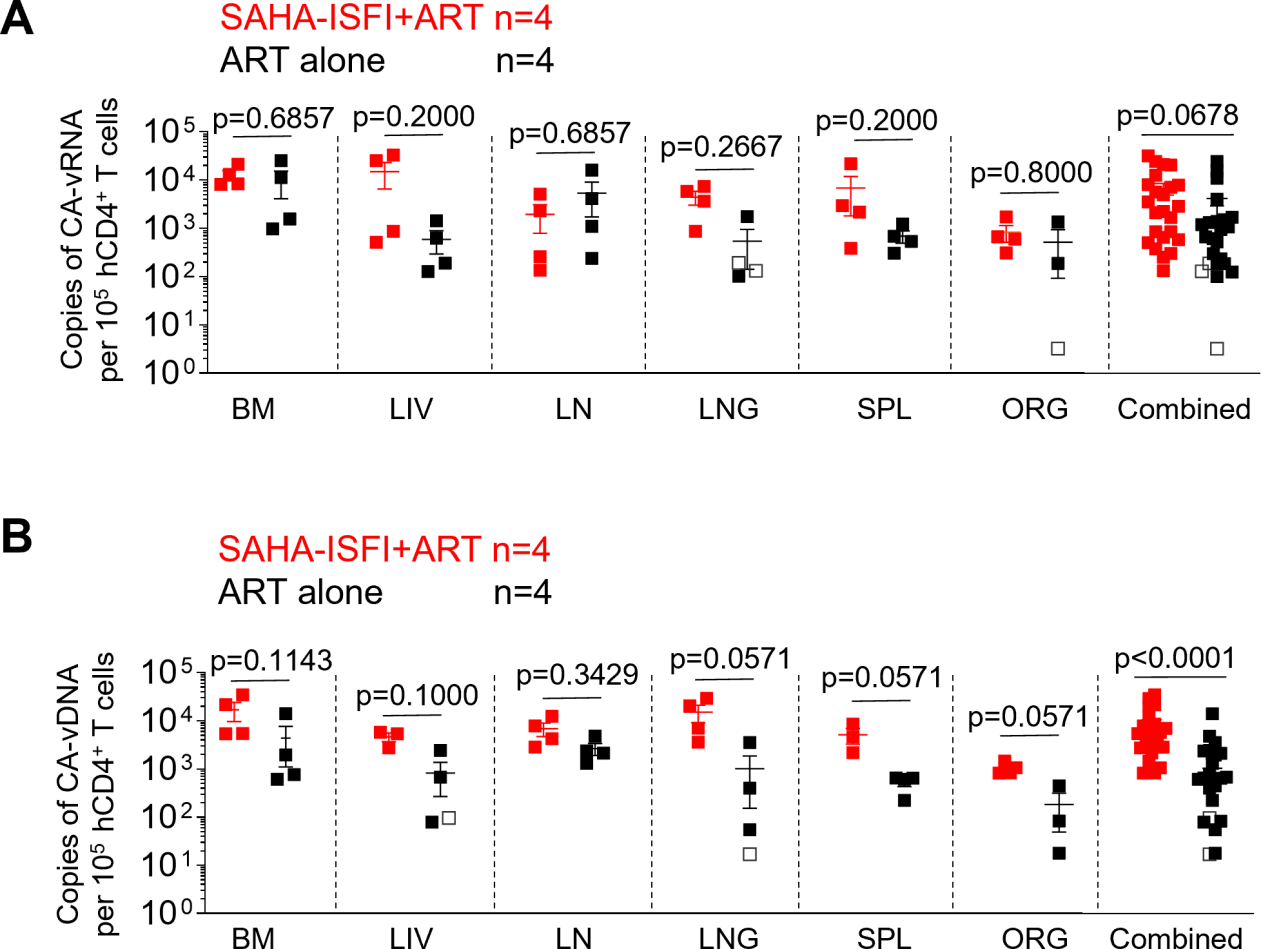
SAHA-ISFI treatment at the same time of ART initiation results in an increase in the systemic levels of cell-associated HIV DNA but does not alter the levels of cell-associated HIV RNA in the tissues of HIV-infected ART-treated humanized mice. CA viral RNA (**A**) and DNA (**B**) were detected in the tissues of HIV-infected mice after treatment with SAHA-ISFI plus ART (red, *n* = 4) or ART alone (black, *n* = 4). BM, bone marrow; LIV, liver; LN, lymph nodes; LNG, lung; SPL, spleen, ORG, human thymic organoid. Combined: all individual tissues from all animals graphed together. Open symbols in panels A and B indicate samples with numbers below the limit of detection. Data are expressed as mean ± SEM. Statistical analyses were performed using unpaired two-sided Mann-Whitney *U*-tests. Statistical significance was considered when *P* < 0.05.

To determine the impact of SAHA treatment at the time of ART initiation on the size of the viral reservoir in the tissues of humanized mice, mouse tissues were collected and mononuclear cells from the bone marrow, liver, lymph nodes, lung, spleen, and human thymic organoid were isolated. Levels of cell-associated HIV RNA in the tissues of humanized mice treated with SAHA plus ART and ART alone were compared. Consistent with the results obtained from cells in peripheral blood, no significant differences in the levels of cell-associated HIV RNA were observed between the SAHA plus ART group and the ART alone group in any individual tissue or when all individual tissues within a group were analyzed together (Fig. 3A).

Next, we evaluated the levels of cell-associated HIV DNA in the tissues of humanized mice treated with SAHA plus ART and compared them with the HIV DNA levels in mice treated with ART alone. The levels of cell-associated HIV DNA in all tissues analyzed from mice treated with SAHA plus ART were higher than those observed in the tissues of mice treated with ART alone. However, these differences were not statistically significant (Fig. 3B). When all individual tissues within a group were combined, we observed significantly higher levels (*P* < 0.0001) of cell-associated HIV DNA in the SAHA plus ART group (8,583 ± 2,024 copies; mean ± SEM) compared with the ART alone group (1,658 ± 624 copies; mean ± SEM) (Fig. 3B). A 2.25-fold decrease was noted in the RNA/DNA ratio in animals that received SAHA when compared with animals that only received ART. Collectively, these data demonstrate that SAHA treatment initiated at the time of ART initiation results in an increase in the systemic levels of cell-associated HIV DNA but does not alter the levels of cell-associated HIV RNA in tissues of HIV-infected, ART-treated humanized mice.

### Treatment with SAHA-ISFI is well tolerated in humanized mice

We measured changes in body weight and changes at the site of ISFI injection in HIV-infected ART-treated humanized mice following SAHA-ISFI treatment initiation. There were no significant differences in body weight between the SAHA plus ART group and the ART alone group and no overt signs of local toxicity at the site of injection (Fig. S1A).

We also evaluated the effect of SAHA-ISFI administration on human immune cells in the peripheral blood of HIV-infected ART-treated humanized mice. No significant differences in the levels of hCD45^+^ cells, hCD3^+^ T cells, hCD4^+^ T cells, and hCD8^+^ T cells were observed at any timepoint following treatment initiation between SAHA plus ART-treated mice and mice treated with ART alone (Fig. S1B through E). We next measured T cell activation levels (CD38^+^HLA-DR^+^) in peripheral blood following initiation of SAHA treatment. No significant differences in the levels of CD4^+^ or CD8^+^ T cell activation was observed between the SAHA plus ART group and the ART alone group (Fig. S2A and B).

A comparison of the overall levels of human immune cells isolated from the tissues of humanized mice treated with SAHA plus ART and ART alone was performed. No significant differences were observed in the numbers of human CD45^+^ cells in any tissue analyzed between the SAHA plus ART group and the ART alone group (Fig. S3A). Additionally, no significant differences were observed in the frequency of hCD3^+^ T cells, hCD4^+^ T cells, and hCD8^+^ T cells between the SAHA plus ART group and the ART alone group (Fig. S3B through D).

Finally, we compared T cell activation levels in tissues between SAHA plus ART-treated mice and mice treated with ART alone and there were no significant differences in the levels of CD4^+^ or CD8^+^ T cell activation (Fig. S4A and B). Together, these results demonstrate that sustained treatment with SAHA does not cause overt adverse effects in HIV-infected ART-treated humanized mice.

### Panobinostat treatment starting at the time of ART initiation does not alter cell-associated HIV DNA and RNA levels in peripheral blood

**Fig 4:**
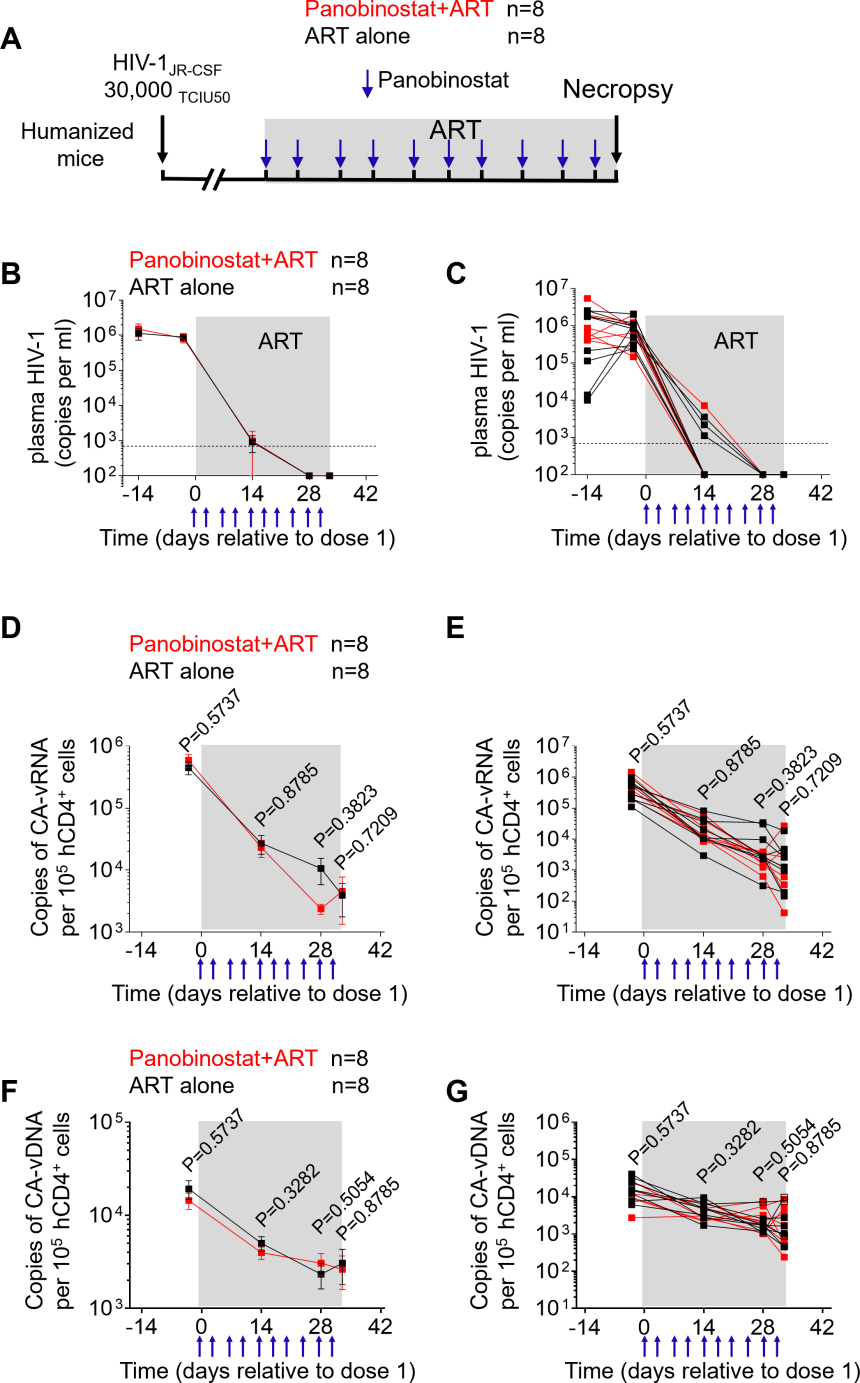
Panobinostat treatment at the time of ART initiation does not alter cell-associated HIV DNA and RNA levels in the peripheral blood cells from HIV-infected ART-treated humanized mice. (**A**) Experimental design. Humanized mice were exposed to HIV on day −28 and then were treated with either panobinostat plus ART or ART alone on day 0. Panobinostat was administered intraperitoneally at 2 mg/kg every 3 or 4 days for a total of 10 doses. ART was administered via FTC (1,500 mg/kg), TDF (1,560 mg/kg), and RAL (600 mg/kg). Longitudinal determination of the plasma viral loads as groups (**B**) and for individual animals (**C**) over the course of the experiment. The plasma viral load limit of detection (693 copies/mL) is indicated by a dashed line. Longitudinal measurements of CA viral RNA as groups (**D**) and for individual animals (**E**) in HIV-infected mice after treatment with panobinostat plus ART (red, *n* = 8) or ART alone (black, *n* = 8). Longitudinal measurements of CA viral DNA as groups (**F**) and for individual animals (**G**) in HIV-infected mice after treatment with panobinostat plus ART (red, *n* = 8) or ART alone (black, *n* = 8). Blue arrows at the bottom indicate the administration of panobinostat. Shaded gray areas depict ART administration. Open symbols in panel G indicate samples with numbers below the limit of detection. Data are expressed as mean ± SEM. Statistical analyses were performed using unpaired two-sided Mann-Whitney *U*-tests. Statistical significance was considered when *P* < 0.05.

Based on the experimental results described above, we decided to evaluate a second histone deacetylase inhibitor with clinical relevance, panobinostat. An experiment was designed to evaluate the effect of panobinostat treatment started at the time of ART initiation on the formation of the HIV reservoir (Fig. 4A). Humanized mice were infected intravenously with HIV-1_JR-CSF_ (30,000 TCIU) on day −28 and then treated with either panobinostat plus ART or ART alone on day 0. Panobinostat was administered intraperitoneally (2 mg/kg) every 3–4 days, as described previously (33). We used this specific dose because it has been previously shown to induce systemic histone acetylation in humanized mice (33). ART was administered via a chow diet as indicated above (29). Plasma viral load was monitored longitudinally following HIV infection. Administration of panobinostat at the same time as ART initiation did not alter plasma viral load decay kinetics (Fig. 4B and C).

To evaluate the impact of panobinostat plus ART treatment on the size of viral reservoir, cell-associated HIV RNA and DNA levels were measured longitudinally in the peripheral blood of humanized mice. No significant differences in the levels of cell-associated HIV RNA were observed between the panobinostat plus ART group and the ART alone group at any timepoint analyzed (Fig. 4D and E). Furthermore, no significant differences in the levels of cell-associated HIV DNA were observed between the panobinostat plus ART group and the ART alone group at any timepoint following treatment initiation (Fig. 4F and G). Additionally, at necropsy the RNA/DNA ratios in the animals receiving panobinostat plus ART and ART alone were 1.74 and 1.28, respectively. Collectively, our results demonstrate that co-administration of panobinostat at the time of ART initiation does not alter cell-associated HIV RNA or DNA levels in peripheral blood.

### Panobinostat treatment initiated at the time of ART initiation does not alter systemic levels of cell-associated HIV RNA and DNA in tissues

**Fig 5:**
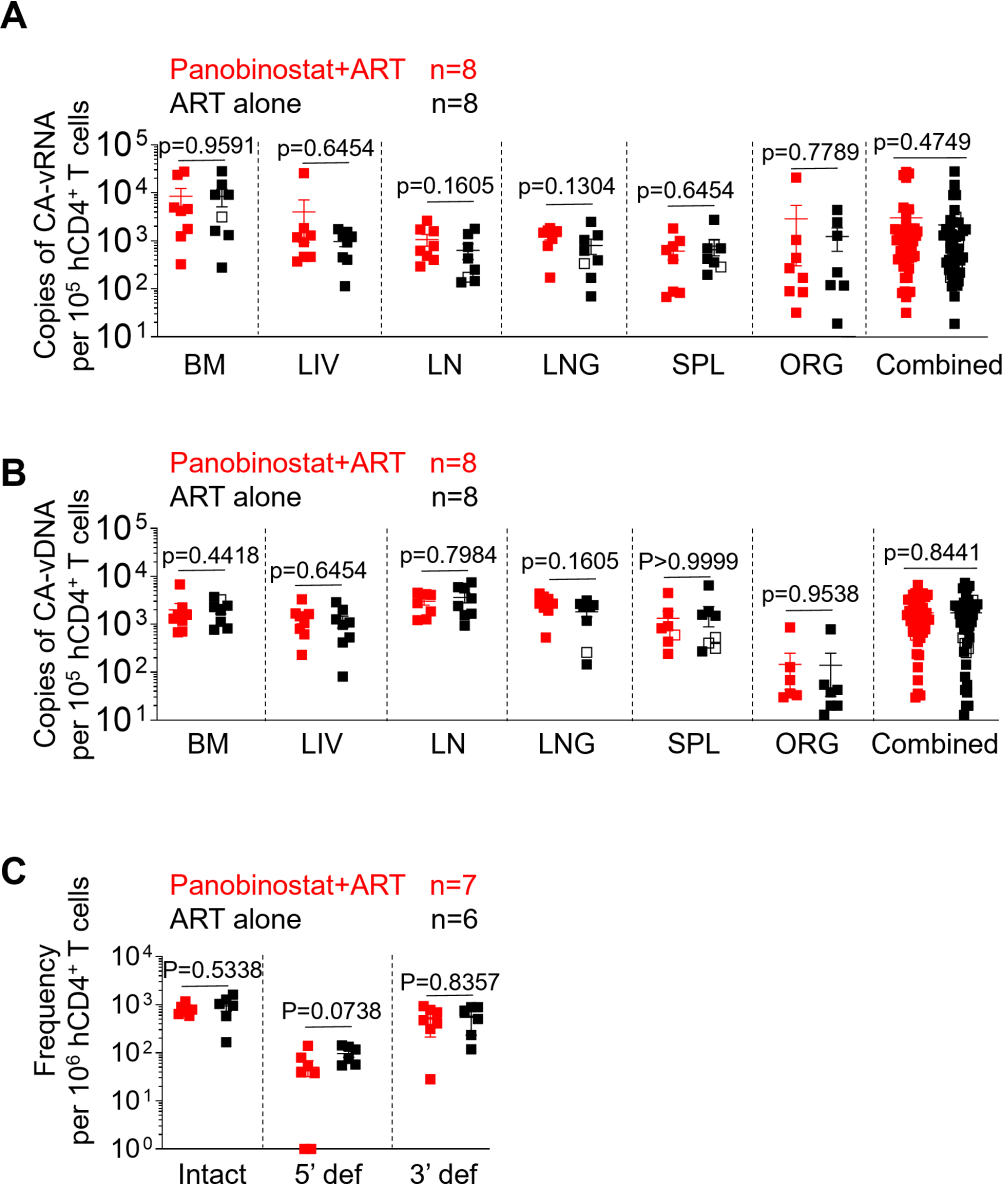
Panobinostat treatment at the time of ART initiation does not alter systemic cell-associated HIV DNA and RNA levels in the tissues of HIV-infected ART-treated humanized mice. (**A**) Cell-associated HIV RNA and (**B**) DNA levels in the tissues of HIV-infected mice after treatment with panobinostat plus ART (red, *n* = 8) or ART alone (black, *n* = 7). Combined: all individual tissues from all animals graphed together. (**C**) The frequency of intact, 5’ def and 3’ def HIV-1 proviruses per million CD4^+^ T cells was detected in the tissues of HIV-infected mice after treatment with panobinostat plus ART (red, *n* = 7) or ART alone (black, *n* = 6). BM, bone marrow; LIV, liver; LN, lymph nodes; LNG, lung; SPL, spleen, ORG, human thymic organoid. Open symbols in panels A and B indicate samples with numbers below the limit of detection. Data are expressed as mean ± SEM. Statistical analyses were performed using unpaired two-sided Mann-Whitney *U*-tests. Statistical significance was considered when *P* < 0.05.

Mononuclear cells from the bone marrow, liver, lymph nodes, lung, spleen, and human thymic organoid of mice were isolated at necropsy to determine the systemic effect of panobinostat and ART co-administration on the size of the HIV tissue reservoir. Levels of cell-associated HIV RNA in the tissues of HIV-infected humanized mice were compared between the panobinostat plus ART group and the ART alone group. No significant differences in the levels of cell-associated HIV RNA were observed between panobinostat plus ART-treated mice and mice treated with ART alone when tissues were analyzed individually or when all tissues within a group were combined (Fig. 5A).

Next, a comparison of the tissue levels of cell-associated HIV DNA between the panobinostat plus ART group and the ART alone group was performed. There were no significant differences in the levels of cell-associated HIV DNA between treatment groups when tissues were analyzed individually or when all tissues within a group were combined (Fig. 5B). Additionally, at necropsy, the RNA/DNA ratios in the animals receiving panobinostat plus ART and ART alone were 1.73 and 1.22, respectively. These results indicate that co-administration of panobinostat starting at the time of ART initiation does not alter cell-associated HIV RNA or DNA levels in tissues.

We next performed an intact proviral DNA assay (IPDA) to determine the size of the intact proviral HIV reservoir in the tissues of humanized mice. Consistent with the observed levels of cell-associated HIV DNA, no significant differences in the frequency of intact proviruses were seen between the panobinostat plus ART group and the ART alone group (Fig. 5C). Together, these results show that under our experimental conditions, panobinostat treatment started at the same time of ART initiation does not result in differences in HIV RNA or DNA levels.

### Treatment with panobinostat is well tolerated in humanized mice

Mouse body weight was monitored over time following treatment initiation. Changes in body weight were not significant between mice treated with panobinostat plus ART and ART alone, except on day 14 post treatment initiation, when there was a small but significant loss in body weight observed in panobinostat plus ART-treated mice compared with mice treated with ART alone (Fig. S5A).

To evaluate the potential toxicity of panobinostat on human immune cells, we measured hCD45^+^ cell, hCD3^+^ T cell, hCD4^+^ T cell, and hCD8^+^ T cell levels in the peripheral blood of humanized mice over time. No significant difference was observed between panobinostat plus ART-treated mice and mice treated with ART alone in the frequency of hCD45^+^ cells at any timepoint, except on day 14, when a significantly lower level of human cells was observed in the ART alone group (Fig. S5B). Neither treatment group displayed any significant changes in the frequency of hCD3^+^ T cells, hCD4^+^ T cells, and hCD8^+^ T cells at any timepoint following treatment initiation (Fig. S5C through E). We longitudinally measured T cell activation in peripheral blood following panobinostat treatment initiation. Again, no significant differences in the levels of CD4^+^ or CD8^+^ T cell activation were observed between mice treated with panobinostat plus ART and ART alone (Fig. S6A and B).

Next, total human CD45^+^ cells in the tissues of humanized mice were compared between mice treated with panobinostat plus ART and mice treated with ART alone. No significant differences were observed in the numbers of human CD45^+^ cells in any tissue analyzed (Fig. S7A). There were also no significant differences observed in the frequency of hCD3^+^ T cells, hCD4^+^ T cells, and hCD8^+^ T cells between mice treated with panobinostat plus ART and ART alone (Fig. S7B through D).

Finally, we compared CD4^+^ and CD8^+^ T cell activation levels at necropsy between mice treated with panobinostat plus ART and ART alone. No significant differences were observed in the levels of CD4^+^ T cell activation in any tissue analyzed between panobinostat plus ART-treated mice and mice treated with ART alone in any tissue analyzed (Fig. S8A). Significant differences were observed only in the level of CD8^+^ T cell activation in the bone marrow and spleen (Fig. S8B). Together, these results demonstrate that panobinostat administration in combination with ART does not have overt toxic effects on HIV-infected ART-treated humanized mice.

## DISCUSSION

HIV cure strategies have mainly focused on either the elimination of the viral reservoir by a “shock-and-kill” approach or on the prevention of viral reactivation from deep latency by a “block-and-lock” approach (3–6, 21, 30, 31, 34, 35). However, little progress has been made with these two approaches. Recent studies indicate that the HIV latent reservoir is more dynamic than previously thought and that after ART, much of the HIV reservoir that persists is genetically linked to viruses that begin circulating within 1 year of ART initiation (17–19). This finding suggests that novel strategies might be developed for deployment at the time of ART initiation, to prevent the establishment of much of the viral reservoir.

HDACs facilitate the deacetylation of histone proteins and thus lead to HIV transcriptional repression. In animal models as well as clinical trials, HDAC inhibitors have been broadly shown to induce HIV expression. A recent study indicates that HDACs play a critical role in initiating latency and that in an *ex vivo* model system of HIV latency, HDAC inhibitors could effectively reduce the frequency of actively infected cells transitioning to the latent state (22). This finding suggests that co-administration of HDAC inhibitors during the initiation of ART may reduce the frequency of latently infected cells that persist on ART.

SAHA and panobinostat are both safe and well tolerated in clinical trials (3–5, 21), and we confirmed that neither SAHA nor panobinostat induced any overt adverse effect on HIV-infected ART-treated humanized mice. However, the relatively short half-life of SAHA in mice compared with humans hampers its use in this model. To achieve sustained drug release, SAHA was formulated as a long-acting injectable *in situ* forming implant. In the current study, we achieved sustained release of SAHA (34.5 ± 5.5 ng/mL; mean ± SEM) from the ISFI into serum for 3 weeks. Similar concentrations of SAHA after a single oral dose in mice dramatically induced systemic H3 acetylation (36). A longer release period was achieved *in vitro* than *in vivo* in the current study. This is consistent with previous observation that PLGA matrix degradation is faster *in vivo* than *in vitro* in a buffer at a physiological pH and temperature (37). In addition, the *in vivo* degradation rate of PLGA implant was significantly faster than *in vitro* one due to an autocatalytic effect of the accumulated acidic degradation products on the medium surrounding the implants (38). Therefore, the difference in SAHA release rate between *in vivo* and *in vitro* is probably due to the different degradation rates of the PLGA implant *in vivo* and *in vitro*. HDAC inhibitors promote acetylation and induce HIV expression; therefore, the use of HDAC inhibitors during ART initiation could potentially slow plasma viremia decay. Ultimately, neither SAHA nor panobinostat treatment altered plasma viral suppression. Additionally, other groups have shown that starting the HDAC inhibitor romidepsin 10 days after ART initiation does not alter viral suppression (11). This study did not provide information regarding the levels of HIV RNA or DNA in tissues. Additionally, in clinical trials, increased plasma viraemia has only been shown with panobinostat and not with SAHA treatment (3–5).

In our study, the administration of SAHA had no effect on plasma or cell-associated HIV RNA levels but it resulted in higher levels of cell-associated HIV DNA in the peripheral blood and tissues of humanized mice. The difference in the levels of cell-associated HIV DNA between the SAHA-ISFI plus ART group and the ART alone group became larger from day 14 (*P* > 0.9999) to day 30 (*P* = 0.0635). SAHA concentrations were detectable up to day 21 and diminished between day 21 and day 28. Although SAHA was undetectable between day 30 to day 43, it is important to note that in a clinical trial, cell-associated unspliced HIV RNA remained significantly elevated 70 days after the last oral dose of SAHA, although the levels of histone acetylation returned to baseline immediately following cessation of drug (4), indicating that SAHA led to a long-term response. The increase in the levels of cell-associated HIV DNA in SAHA-treated animals is consistent with previous *in vitro* study showing that SAHA enhances the susceptibility of primary CD4^+^ T cells to HIV through the inhibition of the cytoplasmic class IIb HDAC6 (39). In addition, impaired NK cell function and reduced virus-specific cytotoxic CD8^+^ T cell responses may also contribute to increased levels of cell-associated HIV DNA after SAHA treatment (40, 41). A decrease in RNA/DNA ratio in SAHA-treated animals compared with control animals may be due to an expansion of infected cells containing either defective virus not giving rise to transcription or virus in a deep latency state.

In contrast to SAHA, co-administration panobinostat through the course of the experiment did not alter levels of cell-associated HIV DNA in our study. This is consistent with previous finding that demonstrating panobinostat does not significantly alter HIV replication in peripheral blood mononuclear cells and CD4^+^ T cells (42). SAHA and panobinostat have different effects on HIV replication *in vitro* even though both are pan-HDAC inhibitors.

Our results demonstrate that in contrast to previously published *in vitro* results suggesting that administration of SAHA or panobinostat could reduce the establishment of HIV latency, *in vivo* administration of histone deacetylase inhibitors at the same time of ART initiation did not measurably reduce the size of the HIV reservoir.

## MATERIALS AND METHODS

### Preparation of a long-acting formulation of SAHA

The SAHA formulation was prepared using ISFI technology, as previously reported (24). Briefly, SAHA was dissolved in dimethyl sulfoxide (DMSO) at the maximum saturated concentration (365 ± 6 mg/mL; mean ± SEM). Then, a low-molecular weight (15 kDa) acid ending PLGA (Resomer RG 502 H, Sigma-Aldrich) was added at a 1:1 mass-to-solvent ratio. The mixture was incubated at room temperature in a rotary shaker (8 rpm) for 1 day to dissolve the polymer in the drug solution. The amount of SAHA loaded in the resulting formulation (SAHA-ISFI) was 142 ± 0.6 mg/g (mean ± SEM). Experiments were then conducted to determine the *in vitro* and *in vivo* release properties of the SAHA-ISFI formulation.

### *In vitro* release properties of SAHA-ISFI

*In vitro* release properties of the SAHA-ISFI formulation were evaluated in PBS (pH 7.4), as previously described (24). Briefly, 30 µL SAHA-ISFI was directly injected into 10 mL of PBS. Samples were incubated at 37°C with shaking at 100 rpm. At predetermined timepoints, 1 mL of sample solution was collected and replaced with an equal volume of fresh PBS to maintain sink conditions (drug concentration in the sample < 0.58 mg/mL). SAHA-ISFI implants were collected and dissolved in 1 mL of DMSO on day 28 following administration. The concentration of SAHA in the longitudinally collected samples and the drug concentration remaining in the implants was determined by measuring absorbance at 265 nm using a UV-Vis spectrometer (DS11, Denovix, DE, USA). The cumulative percentage and the release rate of SAHA from ISFI were calculated using equations previously published (24).

### *In vivo* pharmacokinetics of SAHA-ISFI

*In vivo* pharmacokinetics of the SAHA-ISFI formulation were assessed in BALB/c mice. Briefly, 50 µL SAHA-ISFI was subcutaneously injected into the back of shaved, anaesthetized BALB/c mice using 1-mL syringes with 19-gauge needles. Serum samples were collected over time following SAHA-ISFI administration and analyzed via liquid chromatography with tandem mass spectrometry (LC-MS/MS) to measure SAHA concentrations.

### Generation of humanized mice

Humanized mice were generated, as reported previously (29–31, 43). In brief, small fragments of human liver and thymus tissue (Advanced Bioscience Resources) were surgically implanted under the kidney capsule of NOD.Cg-*Prkdc^scid^Il2rg^tm1Wjl^*/SzJ (NSG; The Jackson Laboratory) prior to the transplantation of autologous human CD34^+^ hematopoietic stem cells. The reconstitution of human immune cells in the peripheral blood of humanized mice was monitored longitudinally, as described elsewhere (29–31).

### Production and titration of HIV

Stocks of HIV_JR-CSF_ were prepared and titrated, as described elsewhere (29, 30). Briefly, human HEK293T cells were transiently transfected with plasmids encoding HIV_JR-CSF_ to produce virus stocks. Virus stocks were titrated by performing serial fivefold dilutions in TZM-bl cells (AIDS Research and Reference Reagent Program, Division of AIDS, National Institute of Allergy and Infectious Diseases).

### HIV infection and ART treatment of humanized mice

Humanized mice were exposed to 30,000 tissue culture infectious units HIV_JR-CSF_ via tail vein injection (diluted with plain RPMI medium). ART was administered by feeding HIV-infected animals irradiated Teklad chow diet containing 1,500 mg emtricitabine, 1,560 mg tenofovir disoproxil fumarate, and 600 mg raltegravir per kg chow (Research Diets), as previously described (29–31, 43).

### SAHA and panobinostat administration to HIV-infected humanized mice

SAHA-ISFI (50 µL) was subcutaneously injected into the back of shaved, anesthetized HIV-infected humanized mice. Panobinostat was administered intraperitoneally at a dose of 2 mg/kg every 3–4 days (Fig. 4A), as we previously described (33).

### Mononuclear cell isolation

Mouse tissues were collected, and MNCs were isolated using protocols as described previously (29, 43–46). Briefly, MNCs from the spleen, lymph nodes, and human thymic organoid were obtained by passing the tissues through a 70-µm cell strainer with a 3-mL syringe plunger. Bones were processed with a pestle and mortar and bone marrow was filtered through a 70-µm cell strainer. Lung and liver tissues were cut into small pieces, incubated with collagenase/DNase for 30 min prior to passing through a cell strainer. The interphase was collected after Percoll density centrifugation. Lysis of erythrocytes was performed using ACK lysis buffer, if necessary. Cells were counted and aliquoted for cell-associated HIV DNA and RNA quantification and flow cytometry.

### Plasma viral load, cell-associated HIV RNA, and DNA quantification in humanized mice

Mice were bled longitudinally over the course of infection to quantitate plasma viral loads. Plasma RNA samples and cell-associated HIV RNA samples were extracted using the Qiagen RNeasy Mini Kit and the Qiagen QIAamp viral RNA Mini Kit, respectively. One-step real-time quantitative PCR (RT-qPCR) was performed using primers and a probe specific to HIV *gag*. The sequences of the forward and the reverse primers and the probe were as follows: 5′-CATGTTTTCAGCATTATCAGAAGGA-3′, 5′-TGCTTGATGTCCCCCCACT-3′, and 5′-FAM-CCACCCCACAAGATTTAAACACCAT-GCTAA-Q-3′, respectively, as previously described (29–31, 43). The detection limit for the plasma viral load was 693 copies/mL.

Cell-associated HIV DNA samples were processed using the Qiagen QIAamp DNA Blood Mini Kit. qPCR was performed to determine the HIV DNA copy number. Human gamma globin DNA was detected simultaneously, as previously described (29, 43, 45, 47). The sequences for the forward and reverse primers and probe for the detection of human gamma globin were as follows: 5′-CGCTTCTGGAACGTCTGAGATT-3′, 5′-CCTTGTCCTCCTCTGTGA AATGA-3′, and 5′-FAM-TCAATAAGCTCCTAGTCCAGAC-Q-3′, respectively.

The limits of detection for cell-associated HIV RNA and DNA in qPCR are four and two copies, respectively. The limit of detection for cell-associated HIV RNA and DNA for each undetectable individual sample in qPCR was calculated based on the number of human CD4^+^ T cells available for analysis.

### Human CD4^+^ T cell isolation from humanized mice

MNCs from the liver, lung, spleen, lymph nodes, bone marrow, and 20 million from the human thymic organoid were pooled together from each animal (43). Human CD4^+^ T cells were positively selected by two sequential LS columns using CD4 microbeads (Miltenyi Biotech), according to the manufacturer’s instructions. The purity of the isolated cells was checked by flow cytometry.

### Intact proviral DNA assay

IPDA was performed as previously described (48). DNA was extracted from stored CD4^+^ T cell pellets using the Qiagen QiaAmp DNA Mini Kit. A median of 4.4× (Q1 3.1, Q3 4.8) 10^5^ CD4^+^ T cell equivalents was assessed per sample. The median DNA shearing index was 0.35 (Q1 0.34, Q3 0.39), highly comparable to reported human samples (43, 48, 49). Proviral frequencies were left censored at five copies per million CD4^+^ T cells. DNA samples from buffer-only controls, HIV seronegative human donor CD4^+^ T cells, and HIV amplicon gblock controls were used to set thresholds for positive proviral amplification.

### Multicolor flow cytometric analysis

Peripheral blood samples, MNCs, or purified CD4^+^ T cells were stained with a multicolor flow cytometry panel, as previously reported (29–31). Following preincubation with mouse IgG, cells were incubated with the following antibodies: anti-CD45-V500 (clone HI30, BD Biosciences), anti-CD3-APC-R700 (clone UCHT1, BD Biosciences), anti-CD19-PE-Cy7 (clone SJ25C1, BD Biosciences), anti-CD4-APC-H7 (clone RPA-T4, BD Biosciences), anti-CD8-FITC (clone SK1, BD Biosciences), anti-CD38-APC (clone HB7, BD Biosciences), and anti-HLA-DR-PerCP (clone L243, BD Biosciences).

Anti-human CD45, CD3, and CD19 antibodies were used to characterize total human immune cells, T cells, and B cells, respectively. Anti-CD4 and CD8 antibodies were used to identify CD4^+^ and CD8^+^ T cells, respectively. Activated T cells were defined by the co-expression of CD38 and HLA-DR. Red blood cell lysis was performed on blood samples using BD FACS lysing solution (BD Biosciences). Data were acquired on a BD LSRFortessa instrument and analyzed with BD FACSDiva (version 6.1.3).

### Statistical analysis

All data were analyzed and graphed using GraphPad Prism (version 8.02). Data are presented as mean ± SEM. Statistical significance was considered when *P* < 0.05. No statistical methods were used to the predetermine sample size. Investigators were not blinded to group allocations or when assessing outcomes. We utilized unpaired two-sided Mann-Whitney *U*-tests to determine the statistical significance of the differences between treatment groups.

## Data Availability

All relevant data are within the article and its supplemental files.

## SUPPLEMENTAL MATERIAL

Fig. S1 mbio.01632-24-s0001.tif SAHA-ISFI does not cause body weight loss or significant differences in levels of human cells in the peripheral blood of HIV-infected ART-treated humanized mice.

Fig. S2 mbio.01632-24-s0002.tif SAHA-ISFI does not induce T cell activation in the peripheral blood of HIV-infected ART-treated humanized mice.

Fig. S3 mbio.01632-24-s0003.tif Effect of SAHA-ISFI on levels of human cells in the tissues of HIV-infected ART-treated humanized mice.

Fig. S4 mbio.01632-24-s0004.tif SAHA-ISFI does not induce T cell activation levels in the tissues of HIV-infected ART-treated humanized mice.

Fig. S5 mbio.01632-24-s0005.tif Panobinostat treatment does not cause body weight loss or significant changes in humanization levels in the peripheral blood of HIV-infected ART-treated humanized mice.

Fig. S6 mbio.01632-24-s0006.tif Panobinostat treatment does not induce T cell activation in the peripheral blood of HIV-infected ART-treated humanized mice.

Fig. S7 mbio.01632-24-s0007.tif Panobinostat treatment does not result in significant reductions in the levels of human cells in the tissues of HIV-infected ART-treated humanized mice.

Fig. S8 mbio.01632-24-s0008.tif Panobinostat does not induce significant changes in T cell activation in the tissues of HIV-infected ART-treated humanized mice.

Supplemental text mbio.01632-24-s0009.docx Supplemental table and figure legends.

Supplemental material mbio.01632-24-s0010.xlsx Source data.

ASM does not own the copyrights to Supplemental Material that may be linked to, or accessed through, an article. The authors have granted ASM a non-exclusive, world-wide license to publish the Supplemental Material files. Please contact the corresponding author directly for reuse.
