# Supplementary figures and images for: Analysis of the effect of HDAC inhibitors on the formation of the HIV reservoir
Source: mBio. 2024 Aug 13;15(9):e01632-24. doi: 10.1128/mbio.01632-24 (PMC11389399; doi:10.1128/mbio.01632-24)

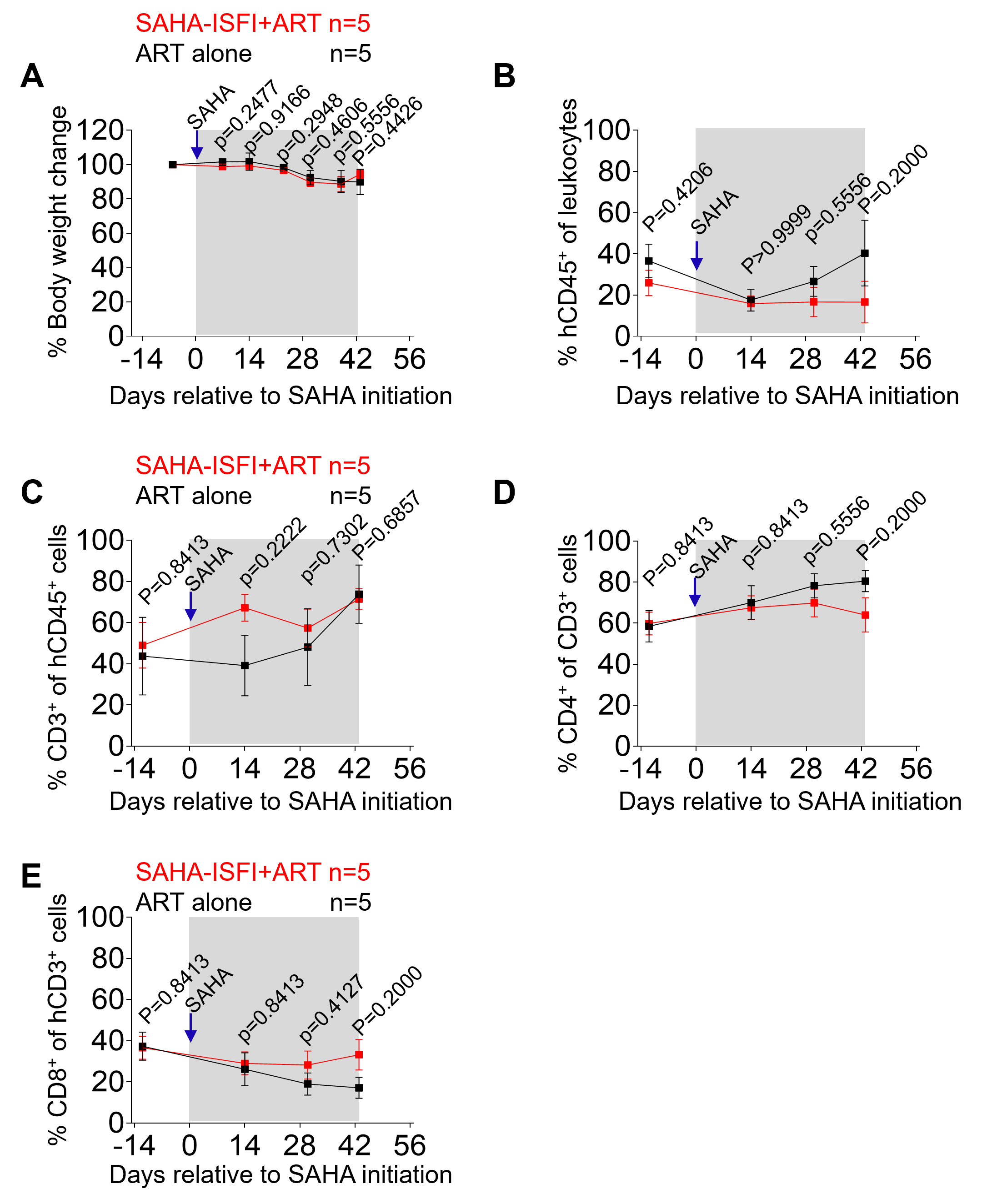

Supplement: Fig. S1 — SAHA-ISFI does not cause body weight loss or significant differences in levels of human cells in the peripheral blood of HIV-infected ART-treated humanized mice. [file mbio.01632-24-s0001.tif]

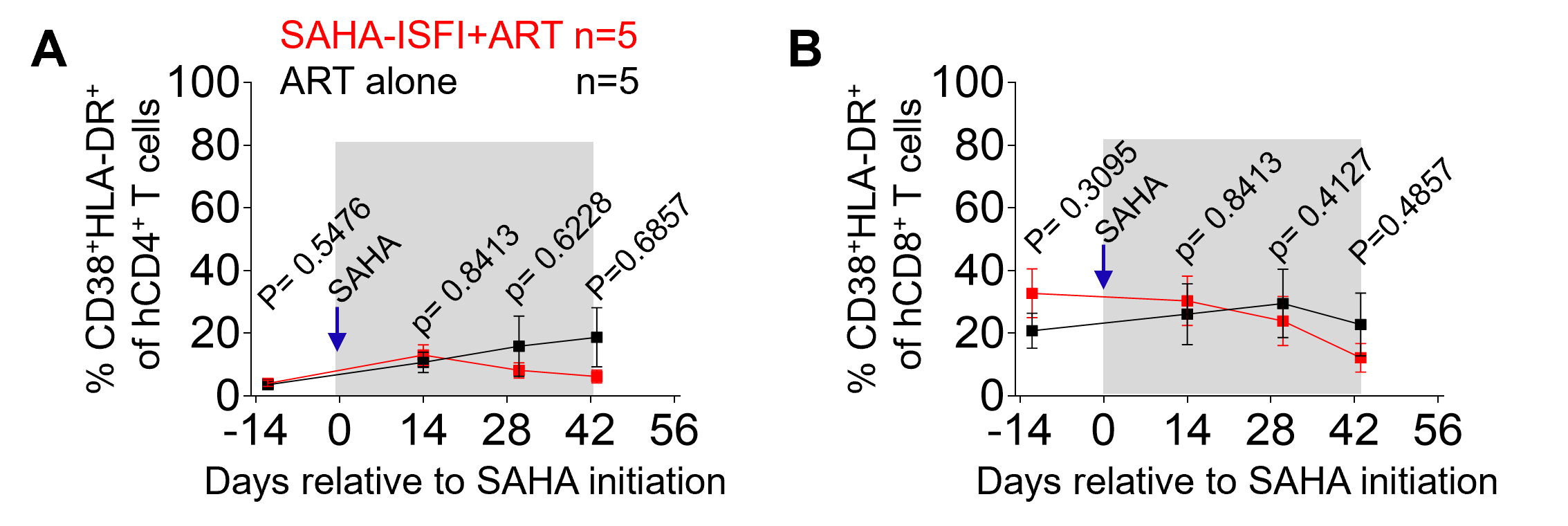

Supplement: Fig. S2 — SAHA-ISFI does not induce T cell activation in the peripheral blood of HIV-infected ART-treated humanized mice. [file mbio.01632-24-s0002.tif]

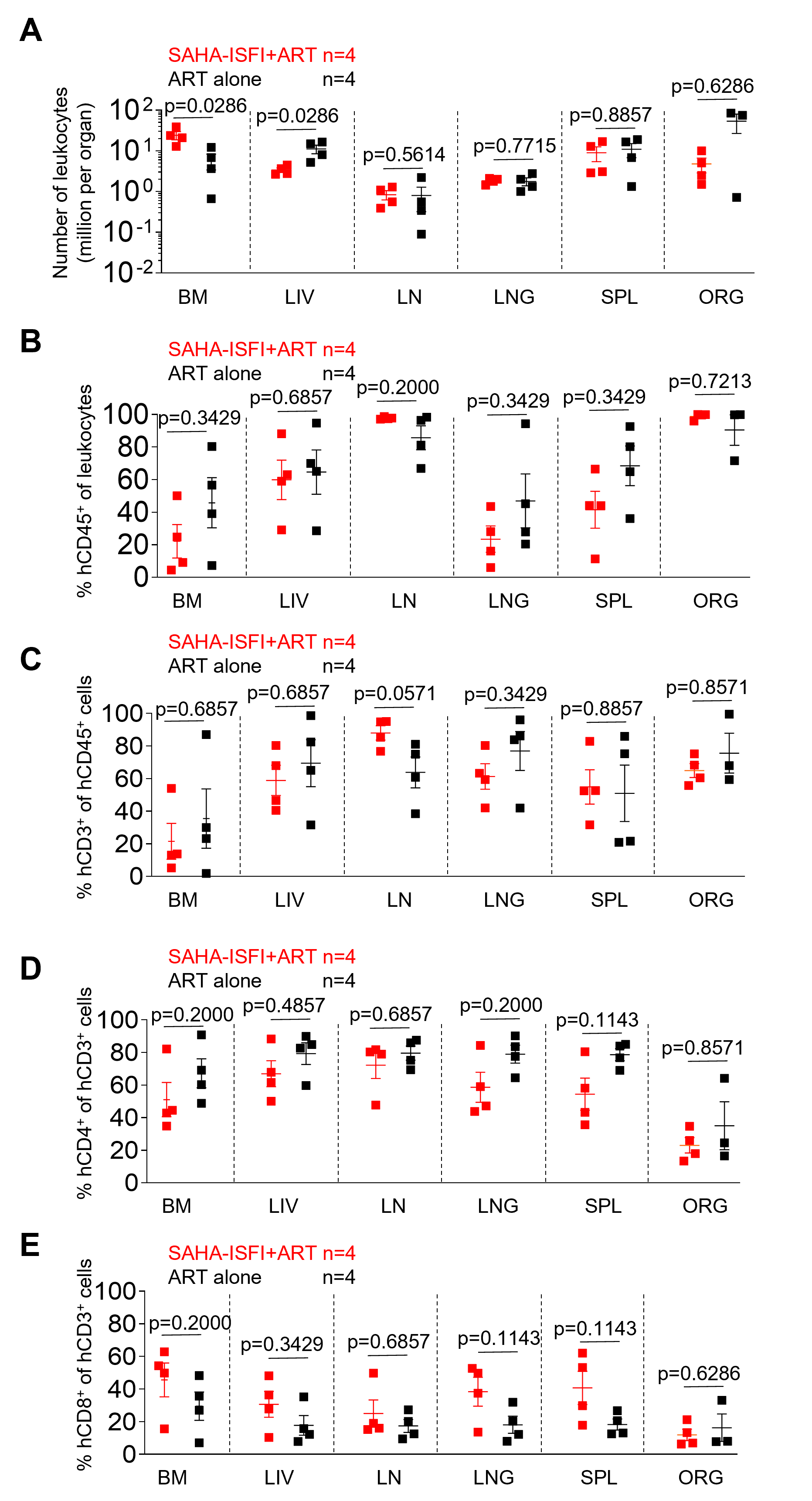

Supplement: Fig. S3 — Effect of SAHA-ISFI on levels of human cells in the tissues of HIV-infected ART-treated humanized mice. [file mbio.01632-24-s0003.tif]

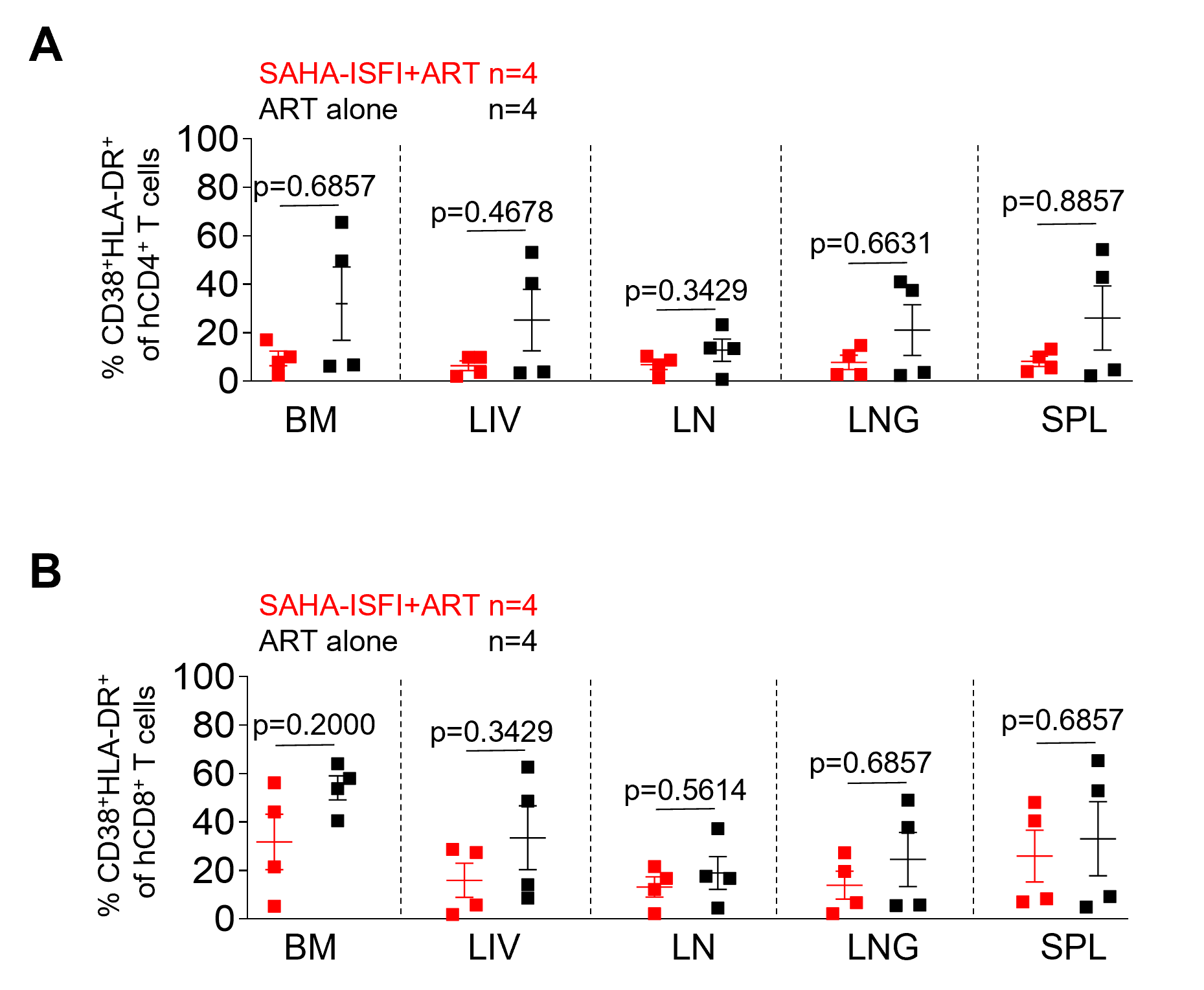

Supplement: Fig. S4 — SAHA-ISFI does not induce T cell activation levels in the tissues of HIV-infected ART-treated humanized mice. [file mbio.01632-24-s0004.tif]

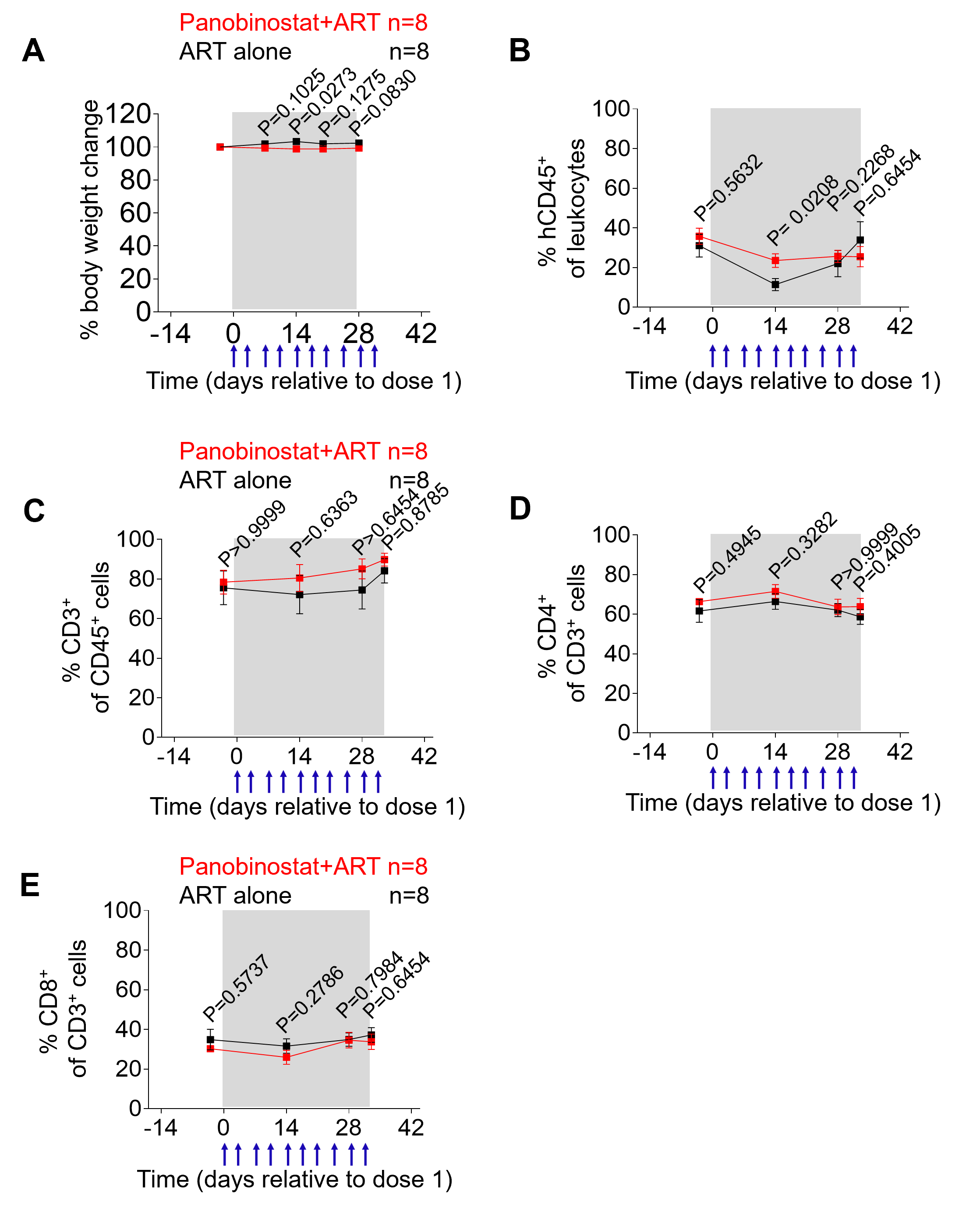

Supplement: Fig. S5 — Panobinostat treatment does not cause body weight loss or significant changes in humanization levels in the peripheral blood of HIV-infected ART-treated humanized mice. [file mbio.01632-24-s0005.tif]

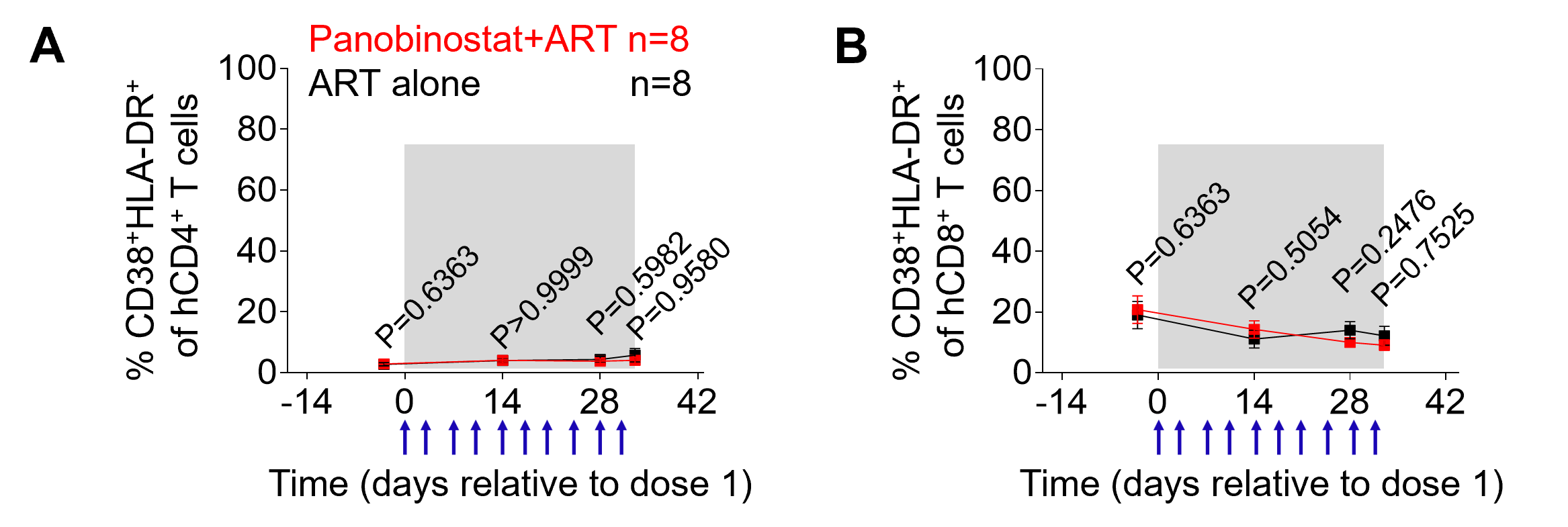

Supplement: Fig. S6 — Panobinostat treatment does not induce T cell activation in the peripheral blood of HIV-infected ART-treated humanized mice. [file mbio.01632-24-s0006.tif]

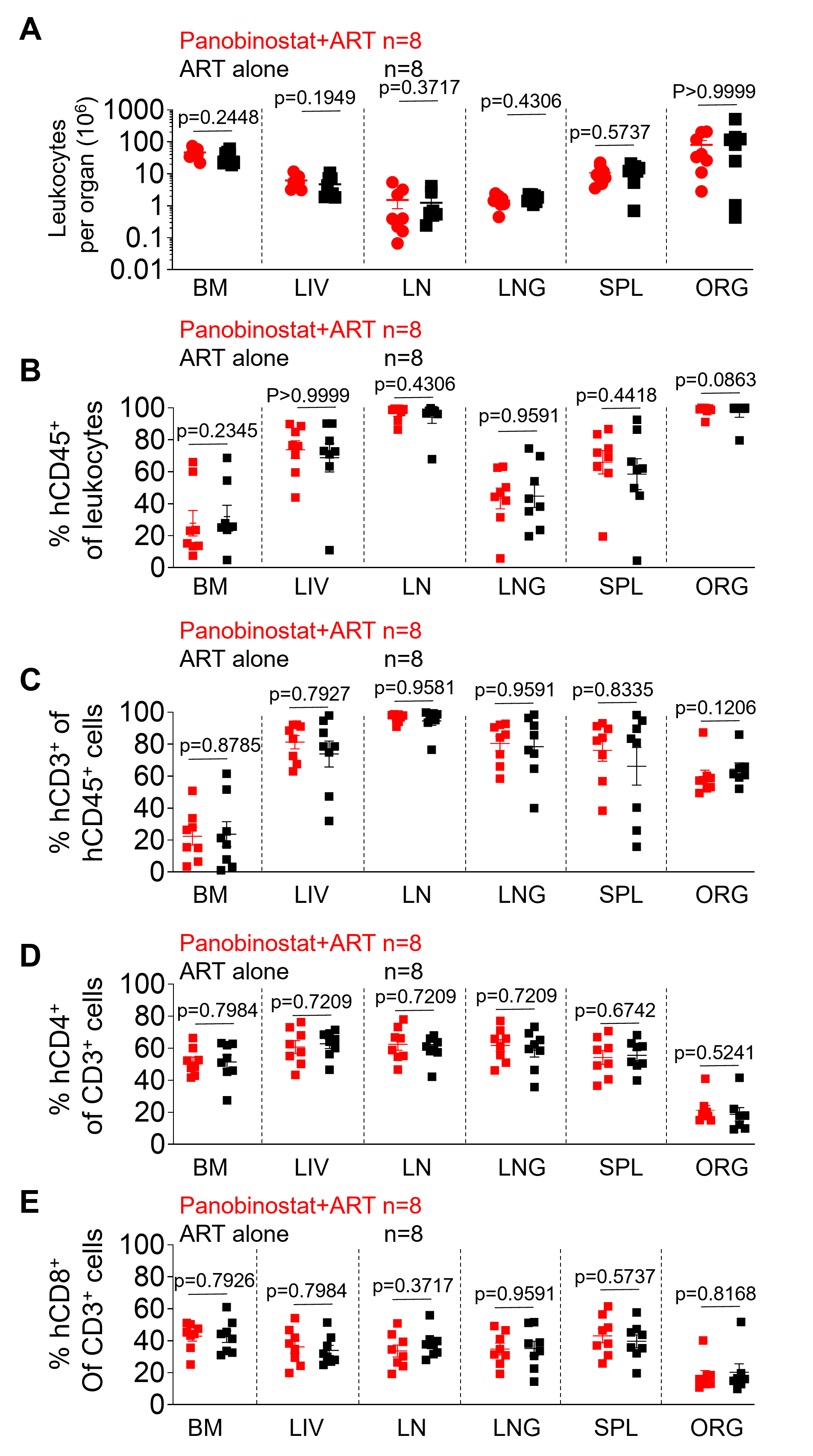

Supplement: Fig. S7 — Panobinostat treatment does not result in significant reductions in the levels of human cells in the tissues of HIV-infected ART-treated humanized mice. [file mbio.01632-24-s0007.tif]

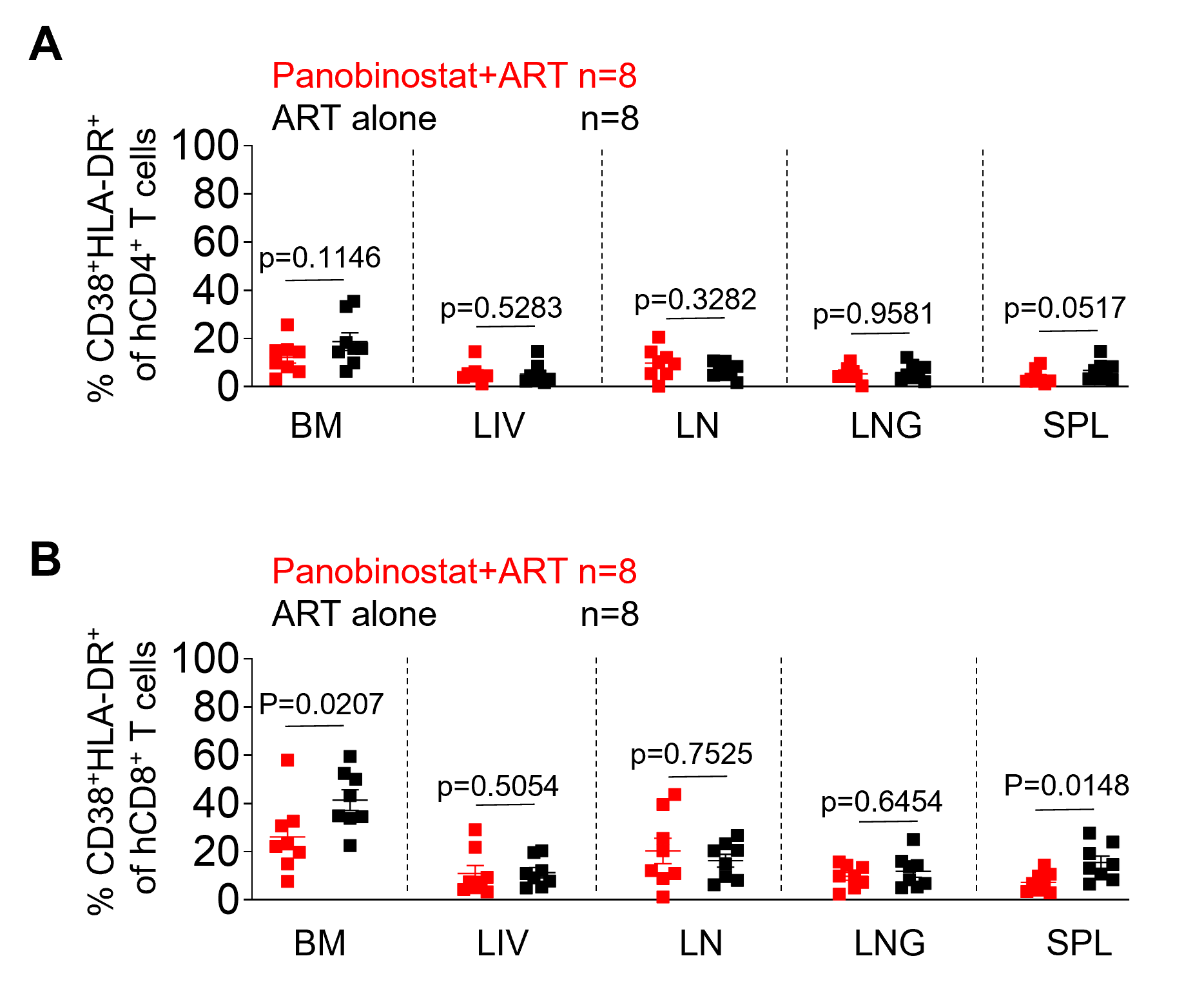

Supplement: Fig. S8 — Panobinostat does not induce significant changes in T cell activation in the tissues of HIV-infected ART-treated humanized mice. [file mbio.01632-24-s0008.tif]
