# Supplementary material for: Analysis of the effect of HDAC inhibitors on the formation of the HIV reservoir
Source: mBio. 2024 Aug 13;15(9):e01632-24. doi: 10.1128/mbio.01632-24 (PMC11389399; doi:10.1128/mbio.01632-24)
Supplement: Supplemental text — Supplemental table and figure legends. [file mbio.01632-24-s0009.docx]

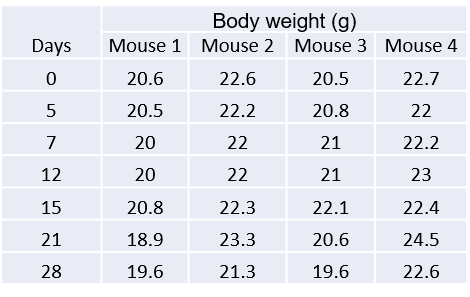


**Supplementary Table 1 Longitudinal monitoring of body weight in BALB/c mice receiving SAHA-ISFI.**

**Figure legends**

**Supplementary Fig. 1 SAHA-ISFI does not cause body weight loss or significant differences in levels of human cells in the peripheral blood of HIV-infected ART-treated humanized mice.** (**A**) Longitudinal monitoring of body weight in HIV-infected mice after treatment with SAHA-ISFI plus ART (red, n=5) or ART alone (black, n=5). Longitudinal flow cytometry analysis the frequency of human CD45^+^ cells (**B**), CD3^+^ T cells (**C**), CD4^+^ T cells (**D**) and CD8^+^ T cells (**E**) in the peripheral blood of HIV-infected mice after treatment with SAHA-ISFI plus ART (red, n=5) or ART alone (black, n=5). Blue arrows indicate the time of SAHA-ISFI administration. Shaded gray areas depict ART treatment. Data expressed as mean ± SEM. Statistical analyses were performed using unpaired two-sided Mann–Whitney U-tests. Statistical significance was considered when *P* < 0.05.

**Supplementary Fig. 2 SAHA-ISFI does not induce T cell activation in the peripheral blood of HIV-infected ART-treated humanized mice.** Longitudinal flow cytometry analysis of activated (CD38^+^HLA-DR^+^) CD4^+^ **(A)** and CD8^+^ **(B)** T cells in the peripheral blood of HIV-infected humanized mice after treatment with SAHA-ISFI plus ART (red, n=5) or ART alone (black, n=5). Blue arrows indicate the time of SAHA-ISFI administration. Shaded gray areas depict ART treatment. Data are expressed as mean ± SEM. Statistical analyses were performed using unpaired two-sided Mann–Whitney U-tests. Statistical significance was considered when *P* < 0.05.

**Supplementary Fig. 3 Effect of SAHA-ISFI on levels of human cells in the tissues of HIV-infected ART-treated humanized mice. (A)** Numbers of hCD45^+^ cells isolated from each organ of HIV-infected mice after treatment with SAHA-ISFI plus ART (red, n=4) or ART alone (black, n=4). Flow cytometric analysis of human CD3^+^ T cells (**B**), CD4^+^ T cells (**C**) and CD8^+^ T cells (**D**) in the tissues of humanized mice after treatment with SAHA-ISFI plus ART (red, n=4) or ART alone (black, n=4). BM, bone marrow; LIV, liver; LN, lymph nodes; LNG, lung; SPL, spleen, ORG, human thymic organoid. Data are expressed as mean ± SEM. Statistical analyses were performed using unpaired two-sided Mann–Whitney U-tests. Statistical significance was considered when *P* < 0.05.

**Supplementary Fig. 4 SAHA-ISFI does not induce T cell activation levels in the tissues of HIV-infected ART-treated humanized mice.** Flow cytometric analysis of activated (CD38^+^HLA-DR^+^) CD4^+^ (A) and CD8^+^ (B) T cells in the tissues of HIV-infected humanized mice after treatment with SAHA-ISFI plus ART (red, n=4) or ART alone (black, n=4). BM, bone marrow; LIV, liver; LN, lymph nodes; LNG, lung; SPL, spleen. Data are expressed as mean ± SEM. Statistical analyses were performed using unpaired two-sided Mann–Whitney U-tests. Statistical significance was considered when *P* < 0.05.

**Supplementary Fig. 5 Panobinostat treatment does not cause body weight loss or significant changes in humanization levels in the peripheral blood of HIV-infected ART-treated humanized mice.** (**A**) Longitudinal analysis of body weight in HIV-infected mice after treatment with panobinostat plus ART (red, n=8) or ART alone (black, n=8). Longitudinal flow cytometry analysis of the frequency of human CD45^+^ cells (**B**), CD3^+^ T cells (**C**), CD4^+^ T cells (**D**) and CD8^+^ T cells (**E**) in the peripheral blood of HIV-infected humanized mice after treatment with Panobinostat plus ART (red, n=8) or ART alone (black, n=8). Blue arrows at the bottom indicate the time of panobinostat administration. Shaded gray areas depict ART administration. Data are expressed as mean ± SEM. Statistical analyses were performed using unpaired two-sided Mann–Whitney U-tests. Statistical significance was considered when *P* < 0.05.

**Supplementary Fig. 6 Panobinostat treatment does not induce T cell activation in the peripheral blood of HIV-infected ART-treated humanized mice.** Longitudinal flow cytometry analysis of activated (CD38^+^HLA-DR^+^) CD4^+^ (A) and CD8^+^ (B) T cells in the peripheral blood of HIV-infected humanized mice after treatment with panobinostat plus ART (red, n=8) or ART alone (black, n=8). Blue arrows at the bottom indicate the time of panobinostat administration. Shaded gray areas depict ART treatment. Data are expressed as mean ± SEM. Statistical analyses were performed using unpaired two-sided Mann–Whitney U-tests. Statistical significance was considered when *P* < 0.05.

**Supplementary Fig. 7 Panobinostat treatment does not result in significant reductions in the levels of human cells in the tissues of HIV-infected ART-treated humanized mice.** (**A**) Numbers of hCD45^+^ cells isolated from each organ of HIV-infected mice after treatment with panobinostat plus ART (red, n=8) or ART alone (black, n=8). Flow cytometric analysis of human CD3^+^ T cells (**B**), CD4^+^ T cells (**C**) and CD8^+^ T cells (**D**) in the tissues of humanized mice after treatment with panobinostat plus ART (red, n=8) or ART alone (black, n=8). BM, bone marrow; LIV, liver; LN, lymph nodes; LNG, lung; SPL, spleen, ORG, human thymic organoid. Data are expressed as mean ± SEM. Statistical analyses were performed using unpaired two-sided Mann–Whitney U-tests. Statistical significance was considered when *P* < 0.05.

**Supplementary Fig. 8 Panobinostat does not induce significant changes in T cell activation in the tissues of HIV-infected ART-treated humanized mice.** Flow cytometric analysis of activated (CD38^+^HLA-DR^+^) CD4^+^ (A) and CD8^+^ (B) T cells in the tissues of HIV-infected humanized mice after treatment with panobinostat plus ART (red, n=8) or ART alone (black, n=8). BM, bone marrow; LIV, liver; LN, lymph nodes; LNG, lung; SPL, spleen. Data are expressed as mean ± SEM. Statistical analyses were performed using unpaired two-sided Mann–Whitney U-tests. Statistical significance was considered when *P* < 0.05.
